# Factors influencing intraoperative blood loss in bimaxillary orthognathic surgery

**DOI:** 10.4317/medoral.27222

**Published:** 2025-08-16

**Authors:** Cihan Topan, Ahmet Emin Demirbas, Fatma Doğruel, Kadir Kaan Ümit, Seher Orbay Yaşlı, Emrah Soylu, Dilek Günay Canpolat

**Affiliations:** 1 Assistant Professor, DDS, PhD, Department of Oral and Maxillofacial Surgery, Erciyes University Faculty of Dentistry, Kayseri, Turkey; 2 Associate Professor, DDS, PhD, Department of Oral and Maxillofacial Surgery, Erciyes University Faculty of Dentistry, Kayseri, Turkey; 3 Assistant Professor, MD, Department of Internal Medicine, Erciyes University Medical Faculty, Kayseri, Turkey; 4 Research Assistant, Department of Oral and Maxillofacial Surgery, Erciyes University Faculty of Dentistry, Kayseri, Turkey; 5 Associate Professor, MD, Department of Oral and Maxillofacial Surgery in Anesthesiology, Erciyes University Faculty of Dentistry, Kayseri, Turkey; 6 Associate Professor, DDS, PhD, Department of Oral and Maxillofacial Surgery, Erciyes University Faculty of Dentistry, Kayseri, Turkey; 7 Professor, MD, Department of Oral and Maxillofacial Surgery, Erciyes University Faculty of Dentistry in Anesthesiology, Kayseri, Turkey

## Abstract

**Background:**

The study aimed to investigate the risk factors that could affect intraoperative blood loss in patients who underwent orthognathic surgery.

**Material and Methods:**

The study included a retrospective analysis of 400 patients who underwent bimaxillary orthognathic surgery. Data on demographic, surgical, and hematological parameters affecting intraoperative blood loss were statistically analyzed.

**Results:**

The mean intraoperative blood loss of male patients was statistically higher than that of female patients (*p* ≤ 0.001). The mean blood loss was higher in patients aged 25 years and older than in patients aged 17-24 (*p*=0.004). Patient weight and duration of surgery were positively correlated with the amount of intraoperative bleeding (r = 0.280 and r = 0.371). Platelet (PLT) count negatively correlated with blood loss (r=-0.213). The MPV/PLT ratio and hemoglobin (HGB) levels were positively correlated with bleeding (r=0.208 and r=0.110).

**Conclusions:**

In orthognathic surgeries, factors such as age, gender, body mass, and duration of surgery were found to be associated with intraoperative blood loss. Males, patients over 24, overweight patients, and prolonged surgical procedures are all significant risk factors for bleeding. In addition, high HGB levels, low platelet count, and low MPV/PLT ratios are also associated with an increased risk of blood loss.

** Key words:**Orthognathic surgery, blood loss, risk factors.

## Introduction

Orthognathic surgery is one of the most preferred methods of maxillofacial surgery for correcting dentofacial problems (1). Surgeons are concerned about significant blood loss during bimaxillary surgery, even though it is now regarded as a rather safe surgical technique. The main cause of intraoperative bleeding in bimaxillary surgery is the dense vascular structure of the maxillofacial region and the difficulty of cauterizing or ligating the relevant vessels. The risk of bleeding during these surgeries remains despite the development of hypotensive anesthesia and the description of numerous surgical techniques aimed at minimizing blood loss (2).

According to reports, orthognathic surgery often results in a blood loss volume of about 400 mL. During surgery, blood, fluid, and electrolyte loss may adversely affect the patient's clinical and hematological parameters. Rarely, individuals who have experienced significant blood loss may require blood transfusions to maintain their bodies in equilibrium (1,3,4). Therefore, surgeons need to know the amount of intraoperative blood loss before a surgical intervention (5). In such cases, it may be necessary to take some precautions for hemostasis before surgery and to be ready for blood transfusion if needed (6).

Numerous factors, including gender, body mass index, weight, blood parameters, surgical duration, anesthetic type, surgical type, and surgeon experience, have been determined to influence intraoperative blood loss in orthognathic surgery (3,7-10). It has not been demonstrated that the amount of intraoperative bleeding is correlated with the outcomes of coagulation screening tests, such as prothrombin time (PT), activated partial thromboplastin time (aPTT), and international normalized ratio (INR), which are commonly administered to patients prior to surgery.. Therefore, before performing orthognathic surgical procedures, surgeons must create a low-cost technique or instrument that may be utilized to predict the amount of intraoperative bleeding (1,11,12). The effect of platelets (PLT) on coagulation has been scientifically proven. Mean platelet volume (MPV) is known as an indicator of platelet activation. Larger and more reactive PLT is associated with higher MPV levels, and research has shown that MPV might alert physicians about possible bleeding risk during preoperative evaluation (13,14). Apart from these, some parameters have been added to the complete blood count (CBC). The neutrophil-to-lymphocyte ratio (NLR) is one of these metrics, and the systemic immune-inflammation index (SII) is another (15-17). This study also used the SII index to estimate the amount of intraoperative bleeding in orthognathic surgery because platelets, an essential component of the hemostatic system, come under its purview. To our knowledge, the SII index has not been used in the literature to predict intraoperative bleeding during bimaxillary surgery.

Although numerous studies exist in the literature on the risk factors influencing the quantity of intraoperative bleeding during orthognathic surgery, the findings about how these factors affect the procedure differ considerably (3,7-9).

This study aimed to investigate the relationship between demographic characteristics, surgical indices, and hematological parameters of orthognathic surgery patients and the amount of intraoperative bleeding.

## Material and Methods

- Study Design and Samples

This study was designed as observational and retrospective clinical research. The study followed the Declaration of Helsinki on medical protocol and ethics, and the Erciyes University Clinical Research Ethics Committee approved it (2024/80). The study carefully examined the hospital records of all patients who underwent orthognathic surgery at Erciyes University Faculty of Dentistry between January 2013 and June 2024. Consent forms were obtained from all patients before surgery.

All patients with complete hospital records, ASA I-II (American Society of Anaesthesiology Classification) classifications, and orthognathic surgery (Le Fort 1 and bilateral sagittal split osteotomy) for dentofacial deformity were included in the study.

The study excluded patients who had bleeding diathesis, were using anticoagulant or antiaggregant medications, had connective tissue disease, had any pathology in the craniofacial region, or had undergone previous surgery in this area. To ensure standardization, patients who underwent genioplasty simultaneously with orthognathic surgery were also excluded from the study.

- Anaesthesia Protocol and Surgical Procedure

The entire surgical procedure was performed under general anesthesia. Patients were given midazolam as premedication to provide preoperative comfort and alleviate anxiety. General anesthesia was induced with an intravenous (IV) dose of 2 mg/kg propofol (Fresenius Kabi, Germany) and 0.6 mg/kg rocuronium (Esmeron; GlaxoSmithKline, UK). A nasotracheal intubation approach was chosen for all patients to meet surgical requirements. Anaesthesia maintenance was achieved with 2% sevoflurane (Sevorane; Abbott, IL) administered in a mixture of 50% oxygen and 50% air. During surgery, controlled hypotensive anesthesia was facilitated by a continuous infusion of remifentanil at a rate of 0.25 μg/kg/min. The infusion was stopped just before extubation. Standard monitoring techniques, including pulse oximetry, electrocardiography, and non-invasive blood pressure measurements, were used throughout the procedure to ensure patient safety and stability. Eight ampoules of 2% articaine and 1:100,000 epinephrine solution (80 mg) diluted 1/1 with 2% articaine and 1:100,000 epinephrine solution (80 mg) were administered to all patients 10 minutes before the surgical procedure for local anesthesia (Ultracain® 2% Ampul, Sanofi Aventis, Istanbul, Turkey). A standard Le Fort 1 osteotomy was performed on the upper jaw, and a modified Obwegeser sagittal split osteotomy (Hunsuck and Epker modification) was performed bilaterally on the lower jaw. During the surgical procedure, care was generally taken to protect the arterial or venous structures in the relevant region. After the upper and lower jaws were placed in their new positions with surgical splints, internal fixation was completed with screws and mini plates (KLS Martin, Tuttlingen, Germany). Incision sites were sutured with 4-0 absorbable suture material (TRUSYNTH®, Coated Polyglactin 910, Healthium Medtech Limited, India). No antifibrinolytic agent or surgical drain was used in any patient. In the postoperative period, the head of the patient's bed was positioned at an angle of 30 degrees. All procedures were performed under the supervision of the anesthesiology and surgical team. The duration between the beginning and completion of each operation was calculated and documented. The amount of intraoperative blood loss was calculated by subtracting the volume of serum solution used during the operation from the volume of fluid collected in the aspirator device. In addition, the volume of blood absorbed by the gauze used in the operation was added to the total amount of blood loss.

- Data Collection

For data analysis, information on the following parameters was collected from the patient files in the hospital archive: 1) patient demographics (age, gender, body weight); 2) surgical indices (duration of surgery, total activation amount, amount of intraoperative blood loss); and 3) hematological indices (hemoglobin, hematocrit, PLT, MPV, MPV/PLT, SII, and NLR) of the patients before the surgical procedure.

The following formulas were used to determine the values of the laboratory indices NLR, SII, and MPV/PLT: MPV/PLT = mean platelet volume/platelet count, SII = (neutrophil count × platelet count) / lymphocyte count, and NLR = neutrophil count/lymphocyte count

- Study variables

Both clinical and laboratory data were used in this study to determine the factors that contribute to intraoperative blood loss in orthognathic surgery. Moreover, clinical factors were divided into surgical and patient-related variables. Patient-related variables were determined as the age, gender, and weight of the patients. Surgical variables included the total amount of activation of the maxilla and mandible and the duration of surgery. Laboratory variables consisted of hemoglobin (HGB), hematocrit (HTC), PLT, MPV, MPV/PLT, SII, and NLR values, including the hematological parameters of the patients.

- Statistical Analysis

The JAMOVI 2.6 package program was used to analyze the research data (The Jamovi Project, 2024, R Core Team, 2024). Descriptive statistical data are given as number (n), percentage (%), mean (x̄), standard deviation (SD), minimum and maximum. The conformity of the data to normal distribution was evaluated by the Shapiro-Wilk test. An independent sample t-test was used to compare paired groups that fit the normal distribution. Spearman correlation analysis was used to determine the relationship between variables. Statistically, *p* <0.05 was considered statistically significant.

## Results

Out of the 712 patient files that were scanned separately from the hospital archives throughout the relevant time, 400 of them satisfied the study's requirements and were thus included in the assessment. Of these patients, 167 (41.8%) were male and 233 (58.3%) were female. Of the study participants, 275 were between 17 and 24 (68.8%), and 125 were over 25 (31.2%). The mean age of the study participants ranged between 17 and 74 years (23.55±6.23). The sociodemographic and operative data of the patients are shown in Table 1.

Surgery was performed on 338 individuals for skeletal class 3 deformity and 62 patients for skeletal class 2 deformity. The mean total activation amount ranged from 3 to 17.50 mm (8.16±2.84), and the mean operation duration ranged from 70 to 420 minutes (241.52±68.39), according to the patient records. The mean total activation amount ranged from 3 to 17.50 mm (8.16±2.84), and the mean operation duration ranged from 70 to 420 minutes (241.52±68.39), according to the patient records. Only one patient required a blood transfusion. This information about the patients is summarized in Table 1. The mean amount of blood loss values of male patients was statistically higher than that of female patients (*p* ≤ 0.001) (Table 2). Moreover, the mean blood loss of the patient group aged 25 years and older was found to be significantly higher than the mean value of the patient group aged 17-24 years (*p*=0.004) (Table 2).

A moderately significant positive correlation was found between the blood loss values of the patients and the values of weight and operation time. It was found that the amount of blood loss increased as the weight of the patients increased (r=0.280). It was also found that the amount of blood loss increased as the operation time increased (r=0.371) (Table 3).

The association between the patients' preoperative hematological parameters and intraoperative blood loss was investigated. A moderately significant negative correlation was found between the PLT count and the amount of bleeding (respectively: r =-0.213). The total amount of blood loss during the procedure was determined to be less in patients with high preoperative platelet count. A moderate positive correlation was found between the amount of bleeding and MPV/PLT values (r=0.208). It was determined that patients with high preoperative MPV/PLT values also had high intraoperative blood loss. A low significant positive correlation was found between preoperative HGB values and blood loss (r=0.110). The amount of blood loss increased in patients with high HGB values before orthognathic surgery. Among the hematological parameters, SII and NLR values were not significantly correlated with the amount of blood loss (r = 0.001 and r = 0.041, respectively) (Table 4).

## Discussion

This study examined the association between the patients' hematological indices, surgical parameters, and demographic characteristics and the amount of intraoperative blood loss during orthognathic surgical operations. According to the current study, a patient's weight, age, gender, duration of operation, and certain hematological parameters are significant predictors of intraoperative blood loss.

Today, orthognathic surgery procedures are defined as safe surgical procedures; however, perioperative blood loss is still considered a major complication (6). To improve patient safety and better address these circumstances, several studies have focused on determining the average expected blood loss volumes in this surgical field (18). Although the estimated blood loss varies widely depending on the type of surgery or various reasons, it is generally accepted to be around 400 ml. (5). Moderate hypotension was achieved in the present study, and the mean amount of blood loss was 357.68±204.81 ml, which is comparable to previous findings. In the literature, there are studies suggesting that age and gender do not affect intraoperative blood loss in orthognathic surgery (1,3,19). However, in a study they conducted, Olsen *et al*. found that women's intraoperative bleeding amounts during orthognathic surgery procedures were lower than men's (7). Similarly, Salma *et al*. reported that the intraoperative bleeding amount of male patients during bimaxillary surgeries was higher than that of female patients (20). In orthognathic surgery, Rummasak *et al*. observed that male patients experienced more intraoperative blood loss than female patients (10). In line with earlier research, it was discovered that male patients had a greater mean intraoperative blood loss than female patients. However, in our study, unlike the literature, the amount of intraoperative blood loss was found to be higher in the elderly patient group (older than 24 years) than in the young patient group (24 years and younger).

Various studies have investigated the relationship between the amount of intraoperative blood loss in orthognathic surgery and the patient's body mass index (BMI). In a retrospective study conducted by Sugahara *et al*., the obese patient group was found to bleed more in bimaxillary surgical operations (19). Thastum *et al*. revealed a negative correlation between BMI and intraoperative relative blood loss. The authors stated that patients with low BMI had higher relative blood loss during orthognathic surgery (3). According to a prospective study by Chrcanovic *et al*., individuals following orthognathic surgery experienced a smaller decrease in hematological parameter values when their weight increased. The authors reported that intraoperative blood loss may be high in the thin patient group. The hemodynamic picture may be affected in such patients, and anemia may develop in the postoperative period (2). Madsen *et al*.'s investigation revealed that the amount of intraoperative bleeding was unaffected by the patient's height, weight, or BMI (1). The present study indicated a moderately positive association between the patients' mass and the amount of intraoperative blood loss. It was demonstrated that as the weight of the patients increased, so did the volume of blood lost during surgery. In this regard, our study's results are consistent with those of Sugahara *et al*.; however, they also run counter to those of several other research studies in the literature (1-3,19). Based on the findings of the present study, it would be advisable to be on alert for potential blood loss during bimaxillary surgeries, particularly in patients who are overweight.

This study's mean operating time was 241.52 minutes, comparable to findings in previous literature. According to these studies, longer operations are associated with greater intraoperative blood loss (2,19). The findings of this study also support the literature. Therefore, the experience of the surgical team, the use of hypotensive anesthesia, and the availability of sufficient equipment and instruments before the treatment are all crucial in reducing the duration of the operation.

Few studies examine the relationship between the movement of the upper and lower jaws and the amount of intraoperative bleeding in orthognathic surgery. A study by Sugahara *et al*. found that the risk of intraoperative bleeding increased when the upper jaw was positioned more than 4 mm higher in bimaxillary surgical operations (19). A retrospective study by Kim *et al*. found that special care should be taken to reduce bleeding risk when performing a superior repositioning in the posterior region of the maxilla (21). This study measured the total activation amount of the upper and lower jaws as 8.16 mm. The study we performed, however, demonstrated that intraoperative bleeding in orthognathic surgery wasn't influenced by the amount of total activation. In this sense, the total amount of blood loss in the surgical process may be affected by the superior repositioning of the upper jaw rather than the quantitative movement of the upper and lower jaws.

In the literature, there are several studies investigating the relationship between the amount of intraoperative bleeding and various hematological parameters (hemoglobin, hematocrit, platelet count, prothrombin time, partial thromboplastin time, and international normalized ratio) in some surgical operations. Lewen *et al*. reported that a preoperative high hematocrit level may increase the risk of intraoperative bleeding in a group of pediatric patients undergoing surgery for neuromuscular scoliosis (22,23). Patients with high preoperative PLT and MPV/PLT values experienced decreased overall blood loss after the surgery, according to the present research. Patients with high HGB values also showed an increase in intraoperative blood loss. Nevertheless, there was no apparent association between the amount of intraoperative blood loss and the hematological indicators HTC, SII, and NLR. PLT count is the main element responsible for intraoperative bleeding. MPV represents the size of platelets and is an indicator of their functionality. Larger platelets adhere better to the vessel walls than smaller platelets and cluster better in that area (13,24). To estimate intraoperative blood loss, it may be useful to look at the patients' PLT and MPV/PLT values prior to the procedure. This is also supported by the findings of our investigation. The current investigation found no association between blood loss and the SII and NLR levels, but a weak correlation between the pre-procedure HGB value and blood loss. Preoperative SII and NLO measurements may not be helpful in estimating intraoperative blood loss. However, further studies involving larger patient populations are required to make a definitive judgment on the validity of these hematological parameters for blood loss estimation.

This study has some limitations. Firstly, BMI values could not be calculated because the data about the height of the patients included in the study were missing. Therefore, the relationship between BMI and the amount of intraoperative bleeding was not evaluated. Secondly, advanced coagulation tests such as coagulation-specific thromboelastography and thromboelastometry were not utilized in our study.

In conclusion, it has been suggested that the amount of intraoperative blood loss in orthognathic surgery may be associated with the patient's age, gender, body mass, and surgical duration. Patients are prone to experience intraoperative bleeding if they are overweight, older than 24, male, and have had prolonged surgeries. Moreover, hematological characteristics that raise the risk of intraoperative blood loss include high HGB levels, low MPV/PLT ratios, and low PLT counts. In this regard, it is crucial to establish an appropriate preoperative preparation procedure in light of anamnesis, clinical examination results, and laboratory testing before surgery to avoid intraoperative bleeding complications in surgical clinics.

## Figures and Tables

**Table 1:** Sociodemographic and operative variables of the patients (n = 400).

Variables	Mean±SD	Min-Max
Age	23.55±6.23	17.00-74.00
Weight	65.68±13.99	21.00-130.00
Amount of blood loss (ml)	357.68±204.81	80.00-2000.00
Operation time (minutes)	241.52±68.39	70.00-420.00
Total activation amount (mm)	8.16±2.84	3.00-17.50

**Table 2:** The distribution of blood loss quantity data is based on the patients' sociodemographic characteristics (n = 400).

Variables		Mean±SD	TEST
Age	17-24	337.94±164.73	t: -4.555
	≥ 25	401.12±268.80	p=0.004
Gender	Female	319.14±162.13	t: -2.886
	Male	411.46±243.09	p≤0.001

t: Independent Samples t Test.

**Table 3:** Correlation between the amount of blood loss, weight, duration of surgery, and total activation values (n = 400).

Scales	Amount of blood loss
Age	0.057
Weight	0.280*
Duration of surgery	0.371*
Total activation value	-0.009

* p<0.05. Pearson Correlation Coefficient.

**Table 4:** Correlation between the amount of blood loss and preoperative hematological parameters (n = 400).

Hematological parameters	Amount of blood loss
PLT	-0.213*
MPV	0.090
MPV/PLT	0.208*
SII	0.001
NLR	0.041
HGB	0.110*
HTC	0.085

* p<0.05. Pearson Correlation Coefficient.
